# Photopolymerization of Coating Materials for Protection against Carbon Steel Corrosion

**DOI:** 10.3390/ma16052015

**Published:** 2023-02-28

**Authors:** Bo Li, Huibing Yang, Jinhang He, Siwu Yu, Rengui Xiao, Huanhu Luo, Yi Wen, Shengyan Peng, Xia Liao, Daning Yang

**Affiliations:** 1 Electric Power Research Institute of Guizhou Power Grid Co., Guiyang 550002, China; 2 College of Chemistry and Chemical Engineering, Guizhou University, Guiyang 550025, China; 3 Electric Power Research Institute of Hainan Power Grid Co., Haikou 570203, China

**Keywords:** photopolymerization, graphene oxide, polyaniline, anticorrosion coatings

## Abstract

This work demonstrated a workable approach for the synthesis of a re-healing polyaniline-modified epoxy resin coating material via photopolymerization. The prepared coating material exhibited low water absorption, allowing it to be used as an anti-corrosion protective layer for carbon steel. First, graphene oxide (GO) was synthesized through the modified Hummers’ method. It was then mixed with TiO_2_ to extend its light response range. The structural features of the coating material were identified using scanning electron microscopy (SEM), X ray diffraction (XRD), and Fourier-transform infrared spectroscopy (FT IR). The corrosion behavior of the coatings and the pure resin layer were tested by using electrochemical impedance spectroscopy (EIS) and the potentiodynamic polarization curve (Tafel). The presence of TiO_2_ reduced the corrosion potential (E_corr_) toward lower values in 3.5% NaCl at room temperature, which was due to the photocathode of titanium dioxide. The experimental results indicated that GO was successfully compounded with TiO_2_ and that GO effectively improved the light utilization capacity of TiO_2_. The experiments showed that the presence of local impurities or defects can reduce the band gap energy, resulting in a lower Eg for the 2GO:1TiO_2_ composite (2.95 eV) compared to that of TiO_2_ alone (3.37 eV). After applying visible light to the coating surface, the change in the Ecorr value of the V-composite coating was 993 mV and the value of I_corr_ decreased to 1.993 × 10^−6^ A/cm^2^. The calculated results showed that the protection efficiency of the D-composite and V-composite coatings on composite substrates was approximately 73.5 and 83.3%, respectively. More analyses revealed that under visible light, the coating had better corrosion resistance. This coating material is expected to be a candidate for carbon steel corrosion protection.

## 1. Introduction

The world factbook shows that the global cost of corrosion is about 3.4% of the global GDP (2013), of which the industrial corrosion costs account for approximately 57.74% [1,2]. Further research could allow between 15 and 35 percent of corrosion’s global cost to be saved by using corrosion control practices [2]. To extend the life of metallic materials and delay corrosion problems, many methods have been developed, such as new alloys with a higher corrosion resistance, corrosion inhibitors, cathodic protection (sacrificial anodes or semiconductor photoanodes), and the application of hydrophobic coatings [3,4,5]. Effectively reducing the corrosion rate has become the focus of anticorrosion research. Excellent corrosion resistance has always been a significant corrosion concern. On the basis of the self-healing properties of individual organisms, several scholars have given attention to re-healing coatings. 

Several common self-healing coating techniques include: (1) Repair agent application [6,7,8,9]. Sun [6], Li [7], Kong [9], and Brown [10] used a healing agent (tung oil, nonaromatic isocyanate derivatives, or amination modification dicyclopentadiene) to form a self-healing capsule. When a crack occurs in the coating, the corrosion breaks the capsule and the core part is released into the damage, healing and repairing the damage. The reports showed that the structure of the capsule influenced the ductility of the coating. (2) Self-healing material application [11,12,13,14,15]. Hager investigated the main approaches for obtaining self-healing materials, including self-healing metals, self-healing ceramics, self-healing concrete, self-healing asphalt, and self-healing polymers [11]. Huang [14], Mather [15], and Michael [16] reported coatings that can be self-healed through heating or light exposure; when the coating is damaged, the thermally responsive body replenishes the coating defects, repairing the protective function. (3) Corrosion inhibitor application [17,18,19]. Harb [17], Yan [18], and Cruz [19] used corrosion inhibitor behavior to grow insoluble substances or change the metal corrosion potential at the damage site, inhibiting the steel substrate’s corrosion process. The mechanisms of self-healing coatings can be divided into intrinsic regeneration (recovery of the structure through an internal force) and extrinsic regeneration (recovery provided by healing agents, corrosion inhibitors, capsules, hollow fibers, or vascular networks), for which some self-healing activities need an external incentive, such as heat [13], pH changes [20], or light exposure [16].

In addition, Taryba [21] used SVET and SIET to investigate local changes to both the ionic activity and the pH, and found that the acidity and current density were higher in the most corroded areas compared to other places. According to corrosion mechanisms, protective measures should reduce the ionic activity and enhance the survival and adaptability in acidic environments. The antiseptic strategy of a pH response under an acid medium forms a passivation film or liberates healing substances. Although light is currently the cleanest and most prosperous energy, it is rarely used to enhance the corrosion resistance of coatings. The current market mainly uses the sensitivity of substances to light to increase their curing speed. Malucelli [22] and Igor [23] have used the free radical polymerization (FRP) method in coatings to prepare ultraviolet-cured acrylate and cycloaliphatic di-epoxy coatings under a UV stimulant. Nguyen [24] observed that loading organic or inorganic nanoparticles into a resin led to an increase in the conversion of acrylate double bonds and mechanical properties.

Polyaniline (PANI) adopts free radical oxidative polymerization in the process of photopolymerization [25]. PANI has both polymer properties and conductive properties [26]; it has attracted considerable attention as a cheap conductive polymer. Conductive polymers have a passivation effect on metals, can inhibit metal corrosion, and are used in metal corrosion inhibitors [27]. Sun [28], Liu [29], and Perrin [30] reported that when PANI is incorporated into an epoxy resin, the coating has obvious hydrophobicity. The coating surface is uniform and compact, the adhesion is good, and the anticorrosion performance is obviously improved. Liu [29] selected doped vulcanized polyaniline as an epoxy filler. The presence of various hybrid-modified polyaniline compounds is beneficial to the corrosion protection of metals. Studies have found that polyaniline can enhance a coating’s hydrophobicity, which may be due to polyaniline’s redox structure; further, this corrosion protection affects exposed bare steel areas that are electrically coupled to the polyaniline-coated metal [31].

In conventional anti-corrosion coatings, PANI and TiO_2_ have been widely used [32,33,34,35]. Despite this, there is currently limited research on the use of visible-light-polymerized aniline as an anticorrosive coating. At present, many documents have reported the use of semiconductors to catalyze the polymerization of aniline at ultraviolet wavelengths [25,36]. A modified titanium dioxide photocatalyst polymerizes aniline under visible light stimulation [37,38,39,40]. There is a lot of research on photopolymerization, but it generally refers to preparing materials and rarely shows the polymer properties in coatings because of the aniline monomer’s toxicity. Furthermore, the aniline monomer evaporates from the coating, which quickly causes the coating pores to become larger and accelerates metal corrosion. 

At the base of the polyaniline as a corrosion inhibitor, the photopolymerization of aniline monomers is used to improve the coating’s compactness through the self-healing effect after defect formation; improve the hydrophobicity of the coating material; prevent the air and electrolytes from contacting the metal substrate; and achieve the goal of improving the corrosion resistance of the coating. In order to achieve this goal, it is key to ensure the polymerization of aniline in the coating while improving the visible light response range and photocatalytic strength of TiO_2_. Herein, we demonstrated a simple manufacturing method for polymerizing aniline monomers in a coating. The dense network structure of the coating can be seen under a polarized light microscope after visible light excitation polymerization, which can significantly increase the coating’s protective effect of the metal. The interface impedance was enhanced and the electrochemical performance of the coating was improved.

## 2. Experimental Section

### 2.1. Materials

The aniline was provided by the Tianjin Kemiou Chemical Reagent Co. The Shanghai Macklin Biochemical Co. offered the anatase TiO_2_. The Shenzhen Hanhui Graphite Co. delivered natural flake graphite powder (1200 mesh). The Chengdu Jinshan Chemical Reagent Co. Ltd. (Chengdu, China) furnished K_2_S_2_O_8_ (98.3%) and KMnO_4_ (99%). The Chongqing Chuanjiang Chemical Reagent Factory proffered H_2_O_2_ (30%). The Chongqing chuandong chemical (GROUP) CO. (Chongqing, China) provided H_2_SO_4_ (98%) and HCl (>35%). All chemical reagents were used without further purification except aniline, which needed to be distilled to obtain pure aniline.

### 2.2. Composite Synthesis

**Figure 1:**
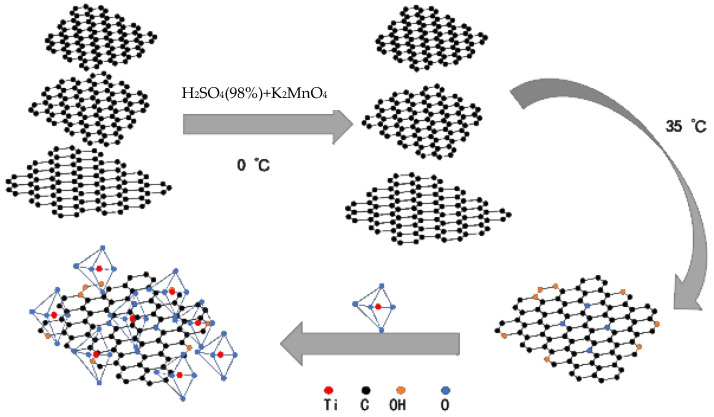
Preparation flow chart of modified TiO_2_ photocatalyst material.

Different amounts of anatase TiO_2_ (A-TiO_2_) were dissolved in a fixed amount of a GO aqueous solution. The GO was obtained using the modified Hummers’ method [41], and the solution was mixed for 1 h in a constant-temperature water bath of 60 ℃ to aid in dispersal. The mixture was then transferred to a high-temperature kettle and allowed to react at 150 °C for 15 h. Then, the product was sufficiently dried in an oven to obtain a modified photocatalyst. Samples of the following mass proportions were made: 1GO:1TiO_2_, 1GO:2.5TiO_2_, and 2GO:1TiO_2_. The preparation flow chart of the modified TiO_2_ photocatalyst material is shown in Figure 1.

Before the photocatalytic polymerization of aniline, the possibility of the often-direct photocatalytic polymerization of aniline (0.2–5 mol·L^−1^) was examined in solutions at different pH values (pH = 2, 4, or 6) in the presence of TiO_2_ under dissimilar irradiation for 12 h. In the same steps as above, the aniline and sulfuric acid were also placed under visible light wavelengths to reduce confusion. After the radiation, the dark sediment was collected and cleaned by centrifugation. It is worth mentioning that the resin used in the experiments was an aqueous self-emulsifiable epoxy resin.

### 2.3. Substrate Preparation

A Q235 carbon steel plate bar was cut into a 10 mm × 15 mm × 5 mm square specimen, sanded with 400-grit sandpaper, cleaned using an ultrasound bath with an absolute ethanol solution, and dried at 40 ℃ in a vacuum oven.

### 2.4. Film Deposition

The distilled aniline monomer, polyaniline particles, and prepared photocatalyst were dispersed in the waterborne epoxy resin (WEP) and compared with the waterborne epoxy emulsion containing only polyaniline particles. The hybrid solutions formed a protective layer of carbon steel by daubing. After the deposition, the coated substrates and the remaining solutions were placed into Teflon cups and placed under darkness and visible light to allow photopolymerization to occur. In order to increase the contrast, we added a waterborne epoxy resin containing only polyaniline particles and named it the composite. The formula was composed of 20 g of bisphenol A epoxy resin E44, 7.28 g of NMP (N-methylpyrrolidone), 2.72 g of n-butanol, 10 g of T31 curing agent, 0.7 g of photocatalyst, 0.3 g of aniline, etc. A ΣIGMA field emission scanning electron microscope from Zeiss, Germany was used.

The filler dispersion of the composite was ultrasonically dispersed for 30 min. The filler was mixed with a water-based epoxy resin (WEP) lotion. After stirring and mixing evenly, a composite compound with a uniform dispersion was obtained. After adding the T31 curing agent, it was coated onto the metal surface.

The photopolymerized solution, dispersed in absolute ethanol, was transferred to a copper plate and observed with a ∑IGMA scanning electron microscope from the Zeiss company, Germany. A UV-3600 scan from a UV-visible spectrophotometer was used to obtain the diffuse reflectance spectra (DRS) by scanning between 200 and 800 nm and using BaSO_4_ as the reference. The X-ray diffraction (XRD) spectra was measured in a range of 2θ from 5° to 70° with Cu Kα radiation, indicating the crystal structure of the synthesized composite material. A Thermo Fisher Scientific Evolution 201 UV-visible spectrophotometer was used to record the UV-visible absorption spectrum. A Nicolet-iS5 Fourier-Infrared Spectrometer from Thermo Fisher Scientific was used to analyze the photopolymerization products’ chemical structure and functional groups. The detection wavelength setting range was 500–4000 cm^−1^. 

The coating thickness was 120 μm. The adhesion of the coatings was tested in accordance with the China National Standard GB/T 9286-1998 (cross-cut test of paints and varnishes). The adhesion grade was level 0 (the cutting edge was complete and smooth, without any shedding). The coating hardness was determined according to the China National Standard GB/T 6739-2006 (pencil method for determination of coating hardness) using Method A—test machine method. The pencil hardness was 4H. 

### 2.5. Electrochemical Assessments

The corrosion protection performance of the aniline photopolymerization hybrid coatings painted on carbon steel was analyzed using electrochemical impedance spectroscopy (EIS) and the polarization curve (Tafel). The tests were conducted in a three-electrode cell comprising the coated carbon steel, a platinum plate, and a saturated calomel electrode. The EIS experiments were performed in the frequency range from 10 kHz to 0.01 Hz, with 10 points per decade and an amplitude of 5 mV. The coating surface was analyzed by scanning electron microscopy (SEM), a polarization microscope, and a digital camera.

The corrosion resistance was tested using electrochemical impedance spectroscopy (EIS) and potentiodynamic polarization, as a function of immersion time, in a 3.5 wt% NaCl solution using a conventional three-electrode cell. The electrochemical test showed the corrosion protection behavior. There were three samples in the experiment: the composite (coated with polyaniline particles), the D-composite (dried in the dark), and the V-composite (dried in visible light). The samples were soaked in a 3.5% NaCl solution for two days to study the corrosion-resistant behavior of the coatings under Tafel polarization.

### 2.6. Polarization

A scanning speed of 5 mV/s and a voltage of ±0.3 V under an open-circuit voltage was used for polarization testing.

## 3. Results and Discussion

### 3.1. Structure and Optical Performance Investigation of Modified Photocatalyst

**Figure 2:**
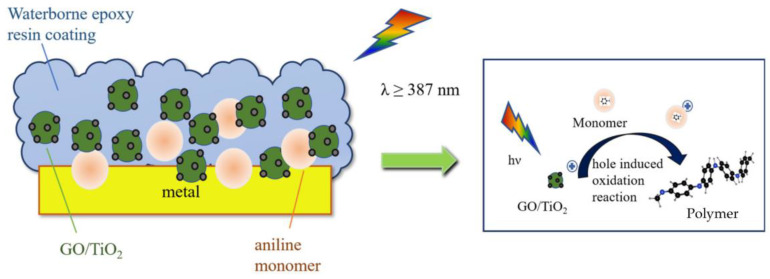
Hole-induced chemistry oxidation process in TiO_2_ photocatalysis.

Inspired by the catalytic mechanism of Guo [42] and Pan [43], Figure 2 shows a schematic diagram of the aniline monomer polymerization mechanism in the coating.

**Figure 3:**
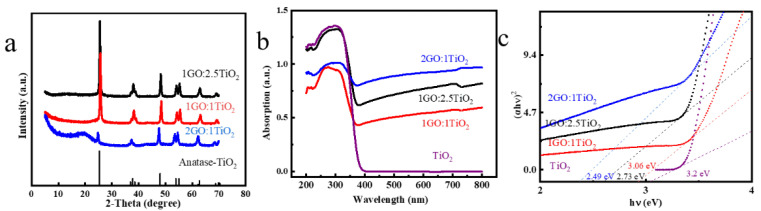
XRD patterns of the samples (TiO_2_, 1 GO:TiO_2_, 1 GO:2.5 TiO_2_, and 2 GO:1 TiO_2_) (**a**); DRS spectra of the catalysts (**b**); and Tauc plots of the samples to determine the band gaps (**c**).

Owing to its physical properties, A-TiO_2_ has a band gap of 3.2 eV and an absorption wavelength of 387 nm. GO was doped into A-TiO_2_ to improve the light utilization efficiency of A-TiO_2_, because the lamellar structure and high-charge mobility of GO can improve the efficiency of photogenerated charge transfers and reduce the consumption of free electrons. XRD was used to assess the crystalline phases of the modified photocatalyst. The XRD results of the composite in different proportions (1GO:1TiO_2_, 1GO:2.5TiO_2_, and 2GO:1TiO_2_) are shown in Figure 3a. The diffraction peaks of the main A-TiO_2_ phase (JCPDS No. 21-1272) are shown. The combination of TiO_2_ and GO did not impair the crystalline structure of TiO_2_, thus removing the effect of the crystalline structure on the catalytic activity. Notably, the diffraction peak in the combined samples conformed to the peak diffraction in the A-TiO_2_ phase. Because A-TiO_2_ covered carbon at the thermostatic water bath mixing, there were no distinctive characteristic diffraction peaks for GO in the XRD patterns. Interestingly, when the doping amount of GO was relatively high, the new diffraction peak appeared around 2θ = 20° in the composite, and the intensity of some diffraction peaks of the A-TiO_2_ phase declined. However, the combined GO diffraction peak with a high GO content did not appear [41]. The GO may have responded at 150℃, which would have diminished certain functional groups of oxides (C-O, C=O, and COOH) and improved the reducibility [44], thereby reducing TiO_2_ [45]. The results show that the doping of a small amount of GO did not cause any change in the structure of TiO_2_.

We used DRS to consider the optical properties of the modified photocatalyst. According to Figure 3b, the semiconductor nature of TiO_2_ led to strong absorption in the range between 200 and 400 nm. There was a specific enhancement in the absorbance from approximately 400 to 800 nm for the composite compared with pure TiO_2_. This is indicative that GO affected the light response of TiO_2_. In addition, we calculated the energy of the band gap with the Tauc plot method (Equation (1)):

(1) $$ah\nu =A{\left(h\nu -E_{g}\right)}^{n/2}$$

The value of n in the above equation decides the band gap. As A-TiO_2_ is an indirect band gap semiconductor, the above formula’s power exponent was two. In addition, Figure 3c shows the picture plotted by (αhν)^2^ versus hν as the vertical and horizontal coordinates. The print illustrates that the *E*g values of TiO_2_ and composites were approximately in the center at 3.25 eV. Furthermore, the *E*g value of the three composites was less than 3.31 eV. The presence of local impurities or defects can reduce the band gap energy [46], resulting in a lower Eg for the 2GO:1TiO_2_ composite (2.95 eV) than for the TiO_2_ alone (3.37 eV). This indicates that GO was successfully compounded with TiO_2_ and that GO effectively improved the light utilization capacity of TiO_2_.

### 3.2. Structural Analysis of PANI

**Figure 4:**
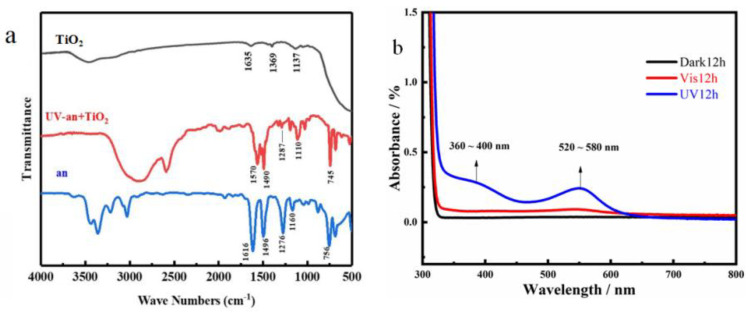
Structural analysis of PANI under different photopolymerization conditions: (**a**) FT-IR spectra and (**b**) UV spectra.

Before modifying the TiO_2_, we used TiO_2_ to promote the polymerization of aniline. The FT-IR spectra of PANI are depicted in Figure 4a. In the process of the photopolymerization of aniline, we chose to dope sulfuric acid to promote photopolymerization. It is worth noting that the black curve in the upper portion of Figure 4a resulted from bare TiO_2_ promoting the polymerization of aniline under visible light. On this curve, peaks at 1137 cm^−1^, 1369 cm^−1^, and 1635 cm^−1^ were observed, and no evidence of aniline monomer polymerization was found. The red curve represents TiO_2_ promoting the polymerization of the aniline monomers in the ultraviolet wavelength range. The prominent characteristic absorption peaks of PANI were all observed to occur in the vicinity of 1570 and 1490 cm^−1^. The absorption peaks at 1570 cm^−1^ and 1490 cm^−1^ can be assigned to the quinoid and benzenoid unit stretching modes (C=C stretching vibration) [47]. They confirmed the polymer’s identity, which was consistent with what has been published in the literature. The results show that pure TiO_2_ has no ability to utilize visible light for polymerization.

The characteristic absorption bands of the intact EB (emeraldine base) form of PANI occur at 336 and 600 nm, respectively [48]. The PANI UV spectra for various light conditions are shown in Figure 4b. There were two transition wavelength bands under UV light. The two absorption peaks of PANI doped with sulfuric acid were at 380 and 560 nm. These are commonly referred to in the 360–400 nm transition of the π–π* bond of the benzene ring structure [48]. The absorption in the visible range, 550 nm, was ascribed to the exciton formation in the quinoid rings [49]. This is in agreement with the IR results in previous work, where pure TiO_2_ catalyzed the polymerization of aniline monomers only under UV light. 

**Figure 5:**
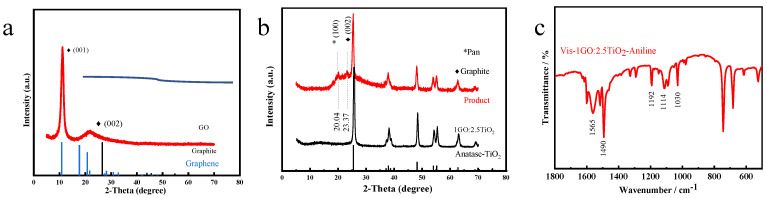
Structural analysis of PANI under visible photopolymerization conditions: (**a**,**b**) XRD patterns and (**c**) FT-IR spectrum.

As a result, doping GO can markedly improve the visible light utilization of TiO_2_. A structural analysis of PANI under visible photopolymerization conditions is shown in Figure 5. The 1GO:2.5Ti expanded the absorption range of visible light for TiO_2_, and the band gap energy was less reduced compared to other ratios of catalysts, so it was selected as the photocatalyst for polyaniline under visible light.

The XRD spectra were measured from 5° to 70°. Figure 5a shows the obvious diffraction peaks of GO at 2θ = 10.08° and 2θ = 21.8°. Compared with carbon (JCPDS No 26-1077) and C60 (JCPDS No 44-0558), the lattice defects of GO prepared using Hummers’ method were slightly greater than those of graphite, and a crystal phase for graphene was found. Figure 5b also shows that traces of the modified catalyst product ideally coincided with the graphite peak (2θ = 23.37°) and Pan’s [47] characteristic peak (2θ = 20.04°). According to the literature report, the amorphous PANI diffusion mostly occurs around 19°, and the characteristic diffraction peak associated with the interplanar spacing between aniline monomers is at approximately 25° [47]. The product from the photopolymerization with 1GO:TiO_2_ had the polymer’s characteristics and was consistent with the literature report. This shows that the action of the catalyst successfully polymerized aniline. The diffraction peak (0 0 2) at 2θ = 23.37°, indicating the distance between graphene layers, was possibly caused by the fact that, during the photopolymerization process and because of agitation, GO was routed out of the TiO_2_ package, and due to the previous thermal reaction, the GO had been reduced and the graphite structure appeared. 

Next, the structure of PANI under visible photopolymerization conditions can be seen in Figure 5c. Compared with pure TiO_2_, although the modified photopolymerization product’s peak absorption regarding the functional group had some deviations, we could still find evidence of successful polymerization. The characteristic peaks that appeared at 1565 cm^−1^ and 1490 cm^−1^ were assigned to the quinoid (Q) rings and benzenoid (B), respectively. This indicates that the doping of GO did effectively improve the visible light utilization of TiO_2_ and helped in the formation of polyaniline from aniline monomers.

### 3.3. Surface Characterization of the Coatings

**Figure 6:**
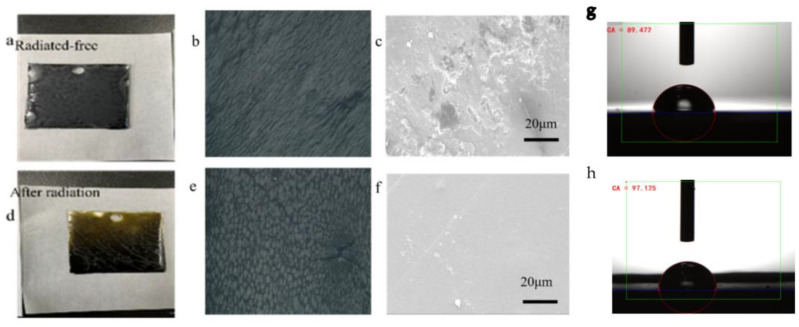
Digital camera, 200 × polarizing microscope, and scanning electron microscope pictures of layers under different light conditions in: (**a**–**c**) the coating dried in the dark; (**d**–**f**) the coating dried under visible light; (**g**) the water contact angle of the epoxy coating dried in the dark; and (**h**) the water contact angle of the epoxy coating dried under visible light.

The scanning electron microscopy (SEM), polarization microscope, and digital camera images of the horizontal surface of the coatings dried under different light conditions are depicted in Figure 6. Figure 6a–c show that the WEP coating surface dried under dark conditions. In the observations of the digital camera, there were no visible pores on the coating surface. The coating surface in Figure 5b is piled up together, probably because the photocatalyst and monomer were not well dispersed within the coating, and this same situation can be seen in Figure 5c, where the material is partially piled up.

Figure 6d–f illustrate the coating dried in the visible light range. In contrast to the dry coating under dark conditions, the surface of the coating under visible light had a smoother surface and a dense lattice structure. The light stimulation improved the surface performance of the WEP coatings. In the figure, it can also be clearly seen that the surface of the layer was relatively flat and had no apparent defects. As shown in Figure 6e, we found an enormous number of columnar substances on the coating surface. This was primarily caused by PANI, which increased the adsorption and film formation of the epoxy–polyamide-hardening materials on its surface and reduced the number of cracks and pores formed [47], which is consistent with previous research findings.

Figure 6g shows the water contact angle of the epoxy coating that was dried in the dark, which was 89.472°; Figure 6h is the water contact angle of the epoxy coating that was dried under visible light, which was 97.125°. The experimental results show that the hydrophobicity was raised when the coating was dried under visible light. This indicates that the compound formed on the coating surface after photopolymerization enhanced the surface hydrophobicity.

Depending on the light condition, we concluded that 1GO:2.5TiO_2_ is a photosensitizer that generates reactive promoting species for polymerization by absorbing energy from irradiated light. Visible light is the condition required to start the reaction; thus, it can convert the energy of light to a chemical potential to render the aniline cations reactive intermediates for reactions [50]. 

### 3.4. Corrosion Protection Evaluation of the Coatings

The cathodic and anodic slopes may be extrapolated from the Tafel polarization curve to obtain parameters such as the corrosion potential (E_corr_) and corrosion current density (I_corr_) [51], as shown in Table 1. Corrosion protection coatings typically have a low corrosion rate (CR), which is equivalent to lower I_corr_ or higher E_corr_ values. The method to calculate the CR is shown in Equation (2) [47]:

(2) $$CR=3270\times \frac{M\left(g\right)\times I_{corr}\left(A\times cm^{2}\right)}{n\rho \left(g/cm^{3}\right)}$$

where *M* is the molecular weight of carbon steel, *I_corr_* is the corrosion current density, *n* is the number of electrons lost in the oxidation process, and ρ is the density of carbon steel.

As can be seen in Table 1, the composite was given a higher corrosion potential (E_corr_ = 35 mV) and the greatest corrosion current (*I_corr_* = 1.194 × 10^−5^ A/cm^2^). This shows that the composite coating itself had a better protective effect on carbon steel and was not easily corroded by corrosive media. When adding the photocatalyst 1GO:2.5TiO_2_ to the composite, the values of E_corr_ and *I_corr_* were reduced to −140 mV and 6.88 × 10^−6^ A/cm^2^, respectively. Li [52] found that the TiO_2_ film in the dark promoted carbon steel corrosion, which caused the E_corr_ to drop. The CR of the D-composite coating decreased, as the *I_corr_* reduction was about 1.73 times less than that of the composite coating. The results show that the modified photocatalyst was beneficial for preventing the corrosion of carbon steel. After applying visible light to the coating surface, the E_corr_ value for the V-composite coating was positively changed to 993 mV. Accordingly, the value of *I_corr_* decreased to 1.993 × 10^−6^ A/cm^2^. The V-composite coating’s CR value was reduced by about 5.99-fold compared with the composite coating, and it was reduced by 3.46 times compared with the D-composite coating, indicating that the coatings involving photopolymerization had a higher corrosion resistance. The improvement in the corrosion resistance of the V-composite coating was mainly derived from passivation, the barrier effects of the doped PANI, photo-generated cathodic protection, and the photopolymerization of the coating surface. On the one hand, promoting aniline polymerization using a photocatalyst can effectively block the pinhole produced by the solvent evaporation of the film-forming solution, make the horizontal plane smoother, and reduce the surface defects of the coating. On the contrary, it could be that as cathodic polarization increases—that is, as concentrated polarization occurs on the cathode—more negative charges are accumulated on the cathode, and the cathode electrode potential shifts in the negative direction by a large amount, thereby reducing the self-corrosion current [28,53]. 

We calculated the protective effectiveness (PE) of the coating assembly using Equation (3) [47]:

(3) $$PE=\frac{I_{corr}\left(Composite\right)-I_{corr}\left(Coated\right)}{I_{corr}\left(Composite\right)}\times 100\%$$

where *I_corr_* (*Coated*) refers to the corrosion current of the D-composite- or V-composite-coated samples. The above equation calculated the protection efficiency of the D-composite and V-composite coatings on composite substrates to be approximately 73.5 and 83.3%, respectively. This result again demonstrates that the introduction of photocatalyst fillers can significantly improve the corrosion resistance of epoxy resin, and that the V-composite coating provides efficient protection after exposure to visible light. The significant increase in the V-composite coating’s corrosion resistance was attributed to the excellent compatibility and dispersibility of the photocatalytic system with the WEP, which reduced the entire coating’s surface deficiencies and promoted the excellent barrier effect of the V-composite in the WEP coating. As can be seen from Figure 6, the V-composite’s surface was very smooth, the reticular formation was closely arranged, and it had a high secondary repairability. 

**Figure 7:**
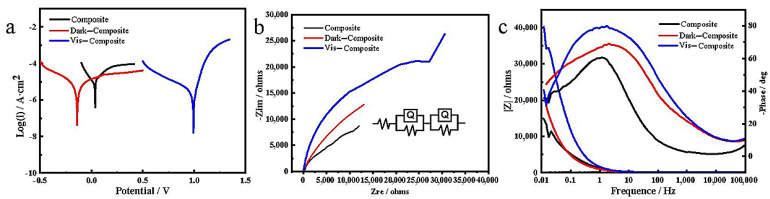
Potentiodynamic polarization curves and EIS studies. (**a**) Tafel plots of composite, D-composite, and V-composite measured in 3.5 wt% NaCl solution with the voltage selection range before and after the open circuit voltage of 300 mv and a scan rate of 5 mV/s; (**b**) Nyquist plots of composite, D-composite, and V-composite measured in 3.5 wt% NaCl solution at the open circuit voltage and frequency ranging from 10 kHz to 0.01 Hz; and (**c**) EIS phases and magnitude of the total impedance of composite, D-composite, and V-composite.

Electrochemical impedance spectroscopy was used to assess the coatings’ corrosion resistance within different light excitations in a 3.5% NaCl solution (Figure 7b,c). To compare the results with those that were not part of the photocatalytic package, EIS measurements were taken for the composite coating. After two days of immersion in the saline solution, the composite was corroded and presented an impedance modulus of 12 kΩ cm^2^ (Figure 7b). To obtain more in-depth insight into the coatings’ electrochemical properties, a fitted equivalent circuit model (ECM) was used to record the EIS curves under different light excitation levels. When the sample was in the immersion phase, a constant ECM was used two times for the analysis, as shown in Figure 7b. An ECM is composed of electrolyte solution resistance (R_s_), coating pore resistance (R_c_), charge transfer resistance (R_ct_), coating capacitance (CPE_c_), and double-layer capacitance (CPE_dl_). The EIS method part of Table 2 shows the detailed corrosion parameters of the ECM fitting.

In the Nyquist plots, the kinetics control corrosion mechanism showed a semicircular characteristic, while the diffusion control showed a linear characteristic [47]. As presented in Figure 7b, the Nyquist plots of the composite, D-composite, and V-composite coatings all expressed high hemispherical arcing, indicating that the corrosion resistance mechanism of these samples is kinetically controlled. Table 1 shows an R_c_ value of 6959 Ω for the V-composite coating, which was 3.40 times and 3.2 times higher than the D-composite’s value of 2048 Ω and the composite’s value of 2120 Ω, respectively. Since the porosity was related to the solvent evaporation of the film-forming solution, this result shows that the photopolymerization on the coating surface could block the pores created by the solvent evaporation of the film-forming solution. The R_ct_ value of the V-composite coating (38.1 kΩ) was also significantly greater than the R_ct_ value of the D-composite coating (20.75 kΩ) and the composite coating (18.2 kΩ). The gradual increase in the coating’s R_ct_ value was due to the formation of a stable and strongly adsorbed passivation film between the coating and the carbon steel. Generally speaking, for a uniform defect-free coating with excellent corrosion resistance, higher R_c_ and R_ct_ values and lower CPE_c_ and CPE_dl_ values are required. Compared with the composite coatings, the CPE values of the D-composite and V-composite coatings gradually decreased, further confirming the difference in the passivation effect of different photopolymerization conditions on carbon steel. The V-composite coating’s excellent protection can be attributed to the material forming a dense coating under visible light polymerization.

The PE of the coatings was also evaluated using the following expression (Equation (4)) [47]:

(4) $$PE=\frac{R_{ct}\left(Coated\right)-R_{ct}\left(Composite\right)}{R_{ct}\left(Coated\right)}\times 100\%$$

where *R_ct_* (Composite) and *R_ct_* (*Coated*) are the composite and coated samples’ charge transfer resistance, respectively. The *PE* of the D-composite and V-composite coatings was estimated from the expression above at approximately 12.3 and 52.2%, respectively. The PE value of the coatings containing photocatalyst fillers was always higher than that of the composite coating, and the V-composite coating was more corrosion resistant. The low frequency (|Z| 0.01 Hz) impedance modulus is also considered a semi-quantitative index of coating corrosion resistance [47]. The Bode plot of the sample (Figure 7c) showed that the WEP coating impedance value exploded after the photoreaction. After two days of immersion, the |Z| 0.01 Hz value of the coating gradually increased from 14,810 Ω for the composite to 18,300 and 40,270 Ω for the D-composite and the V-composite, respectively. Therefore, based on the Tafel and EIS results, we firmly believe that the V-composite coating has a higher corrosion resistance for carbon steel.

## 4. Conclusions

In summary, we demonstrated a photopolymerization method for the preparation of PANI. The V-composite coating’s structure demonstrated that the photocatalyst had a better corrosion protection performance and structural compactness. The surface morphology analysis showed that the photopolymerization method improved the coating’s uniformity, strengthened the coating’s surface structure, and facilitated the dispersion and compatibility of polyaniline in the epoxy resin. The electrochemical corrosion and accelerated immersion tests in a 3.5% NaCl solution showed that the protective effect of the photoreactive coating on carbon steel was more potent than that of the non-photoreactive substance and compound. 

After applying visible light to the coating surface, the change in the Ecorr value of the V-composite coating was 993 mV and the value of *I_corr_* was decreased to 1.993 × 10^−6^ A/cm^2^.

The excellent corrosion resistance of the V-composite coating comes not only from the promotion of aniline polymerization by the photocatalyst, but also from the fact that the photocatalyst made the horizontal surface smoother through the photopolymerization reaction and reduced the surface defects of the coating. The photocatalyst also increased the cathodic polarization, thereby reducing the self-corrosion current. By enhancing the surface structure of the entire coating, the barrier to corrosive media can be increased. 

It can be evidenced that the polymer coating formed by adding non-toxic monomer fillers to WEP can effectively prevent metal corrosion, replace traditional coatings, use light energy effectively, and have potential applications one day.

## Figures and Tables

**Table 1:** Fitted corrosion characterizations for the coating samples after immersion in a 3.5% NaCl solution for 2 days by electrochemical corrosion measurements via the Tafel method.

Electrochemical Corrosion Measurements (SCE Was Employed as a Reference Electrode)				
Sample	Tafel Method			
	E_corr_ _(mV vs. SCE)_	I_corr_ _(A/cm_^2^_)_	C_R_ (mm/year)	PEs (%)
carbon steel	−580	15.65 × 10^−6^	0.147	/
composite	35	11.94 × 10^−6^	0.139	/
D-composite	−140	6.88 × 10^−6^	0.0803	73.5
V-composite	993	1.993 × 10^−6^	0.0232	83.3

**Table 2:** Fitting corrosion characterizations for the coating samples after immersion in a 3.5% NaCl solution for 2 days by electrochemical corrosion measurements via the EIS method, and electrochemical corrosion measurements (SCE was employed as a reference electrode).

Sample	EIS Method					
	R_s_ (Ω cm^2^)	CPE_c_ (F/cm^2^)	R_c_ (Ω cm^2^)	CPE_dl_ (F/cm^2^)	R_ct_ (Ω cm^2^)	PEs (%)
carbon steel	4.017	4.002 × 10^−4^	/	/	0.7677 × 10^−4^	/
composite	8.686	2.009 × 10^−4^	2120	3.992 × 10^−4^	1.82 × 10^4^	/
D-composite	10.95	4.662 × 10^−4^	2048	5.967 × 10^−4^	2.075 × 10^4^	12.3
V-composite	12.46	2.357 × 10^−4^	6959	2.156 × 10^−4^	3.81 × 10^4^	52.2

## Data Availability

Not applicable.

## Abbreviations

Composite, material coated with polyaniline-based composite particles; D-composite, composite material containing a photocatalyst and dried in the dark; EIS, electrochemical impedance spectroscopy; GO, graphene oxide; SEM, scanning electron microscopy; V-composite, composite material containing a photocatalyst and dried under visible light.
